# The Importance of Precursors and Modification Groups of Aerogels in CO_2_ Capture

**DOI:** 10.3390/molecules26165023

**Published:** 2021-08-19

**Authors:** Leila Keshavarz, Mohammad Reza Ghaani, Niall J. English

**Affiliations:** School of Chemical and Bioprocess Engineering, University College Dublin, Belfield, D04 V1W8 Dublin, Ireland

**Keywords:** aerogel, CO_2_ capture

## Abstract

The rapid growth of CO_2_ emissions in the atmosphere has attracted great attention due to the influence of the greenhouse effect. Aerogels’ application for capturing CO_2_ is quite promising owing to their numerous advantages, such as high porosity (~95%); these are predominantly mesoporous (20–50 nm) materials with very high surface area (>800 m^2^∙g^−1^). To increase the CO_2_ level of aerogels’ uptake capacity and selectivity, active materials have been investigated, such as potassium carbonate, K_2_CO_3_, amines, and ionic-liquid amino-acid moieties loaded onto the surface of aerogels. The flexibility of the composition and surface chemistry of aerogels can be modified intentionally—indeed, manipulated—for CO_2_ capture. Up to now, most research has focused mainly on the synthesis of amine-modified silica aerogels and the evaluation of their CO_2_-sorption properties. However, there is no comprehensive study focusing on the effect of different types of aerogels and modification groups on the adsorption of CO_2_. In this review, we present, in broad terms, the use of different precursors, as well as modification of synthesis parameters. The present review aims to consider which kind of precursors and modification groups can serve as potentially attractive molecular-design characteristics in promising materials for capturing CO_2_.

## 1. Introduction

**Figure 1:**
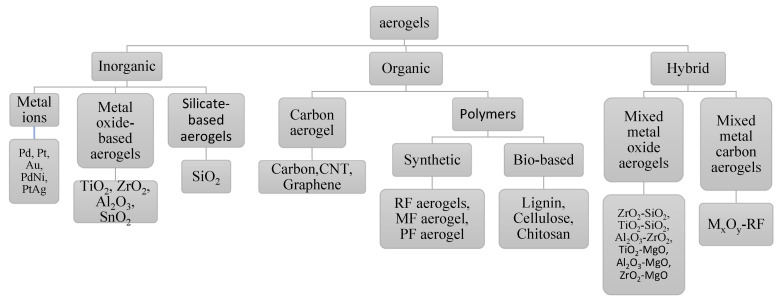
Classification of aerogels.

An aerogel is generally referred to as a material featuring organic, inorganic, or mixed precursors that is prepared by a sol-gel process, in conjunction with drying technology rapidly to extract the liquid in an alcogel, and to replace the liquid to make a three-dimensional and highly porous network. The sol-gel process makes it possible for tailoring the composition and network structure of aerogels. The drying step, as the last stage of aerogels’ preparation, is the most critical step which determines the morphology, porosity, and structural integrity of the final structure [1]. Between various drying methods, such as supercritical drying, ambient-pressure drying, freeze-drying, vacuum drying, and microwave drying, two methods of freeze-drying and supercritical carbon dioxide have emerged as the more common, as they do not damage the underlying microstructure of aerogels [2]. Supercritical drying is the costliest method, which leads to a very open porous sorbent. Therefore, aerogels produced, ipso facto, via supercritical drying are to be directed as most appropriate for use in relation to highly specialized purposes. Ambient-pressure drying provides for a less energy-intensive alternative route, but commonly relies on replacing the original solvent used for gel preparation which is also costly [3,4]. During recent decades, noticeable advancements have occurred in aerogel synthesis, whilst different types of aerogels have been produced [2]. Hence, aerogels can be broadly classified according to their precursors as organic, inorganic and hybrid, as shown schematically in Figure 1. Due to their biosafety, availability, and biodegradability, the focus recently increases on the development of bio-aerogels derived from natural polysaccharides, such as cellulose, chitosan and lignin [5]. Moreover, hybrid aerogels featuring multi-components that show enhanced performance and multi-functional aspects have attracted more interest recently. These aerogels have often conferred additional functionality, such as hydrophobicity, mechanical strength, and catalytic features to original materials.

Owing to the numerous advantages of aerogels (e.g., low density, high porosity, and high surface area), the application for capturing CO_2_ is quite promising. In addition, the flexibility of the sol-gel process, the microstructural form, as well as the composition and surface chemistry of aerogels, can be modified intentionally for CO_2_ capture. To enhance the CO_2_ capacity of porous sorbents at low temperatures, active materials, such as amines, potassium carbonate, and ionic liquids have been added to the surface of aerogels. Amine is one of the most used modification groups which load the porous material to enhance its CO_2_-adsorption capacity. Many researchers have studied the effect of loaded amine to organic and inorganic aerogels on the uptake of CO_2_ by aerogels [6,7,8]. Amine-modified solid adsorbents can be prepared by three techniques, which are classified as (a) physical impregnation (wet impregnation) (b) post-synthesis grafting, and (c) direct condensation [9]. In the impregnation process, amines are incorporated into an adsorbent by van der Waals forces, whereas in the chemical-grafting method an alkanolamine molecule is adsorbed onto a porous surface via reaction with a silanol group [10]. In general, physical impregnation of amines leads to what are called Class I sorbents. For the amine-based adsorbents reported so far, most were prepared by wet impregnation with relatively large amine loading—which constitutes a leading type of Class I sorbent [11]. Covalently bonding amino functional groups to the surface of the support, for example condensing aminotrialkoxysilanes onto a silica surface, lead to the basis of Class II sorbents. Class III sorbents are prepared by polymerizing an amine-rich polymer from the support surface, such as in situ or stepwise polymerisation.

Several factors influence the performance of aerogel in the adsorption of CO_2_, such as temperature, partial pressure of CO_2_, sorbent textural properties, density of the aerogel etc., which have been discussed and analyzed in detail in our previous comprehensive review [2]. Owing to the versatility of the sol-gel process, the pore properties, composition and surface chemistry of aerogels, can be engineered for CO_2_ capture. They can be tailored by the selection of the appropriate precursor and via judicious parameter optimization, such as drying methods. There are some important properties of precursors that require significant consideration for the productivity of aerogels applied in CO_2_ adsorption, such as hydrophobicity and mechanical strength. For example, despite aerogels’ intrinsically light-weight nature, powdered aerogels can block fixed-bed reactors; in such situations, the underlying flexibility and mechanical strength of aerogels are pivotal. Moreover, aerogels featuring precursors of silicon alkoxides and solvents of alcohols are costly and raise environmental issues. To the best of our knowledge, there is no review on the effect of precursors and functionalizing groups on the performance of aerogels in CO_2_ capture. With this clear research gap, the objective of the present review is to discuss a broad range of precursors as well as modification groups used in aerogels for CO_2_-capture applications, and discuss updated aerogels available for commercialization in this field. Within this scope, we attempt to review and critique all of the components (precursors and modification groups) which have been applied for aerogel as adsorbents for CO_2_ capture.

## 2. Inorganic Aerogels

Amine species are modified onto porous supports, such as silica aerogels to boost CO_2_-adsorption capacity. Silica aerogels are the most studied inorganic aerogels for CO_2_ capture, boasting superior properties, such as large specific surface area (500–1200 m^2^∙g^−1^), low density (~0.003 g∙cm^−3^), and high porosity (80–99.8%) [12]. In this context, the application of silica aerogels for CO_2_-capture processes as solid supports has become a focus of great interest [13]. Up to now, most research has mainly focused on the synthesis of amine-modified silica aerogels and their performance as CO_2_ adsorbents [14].

**Figure 2:**
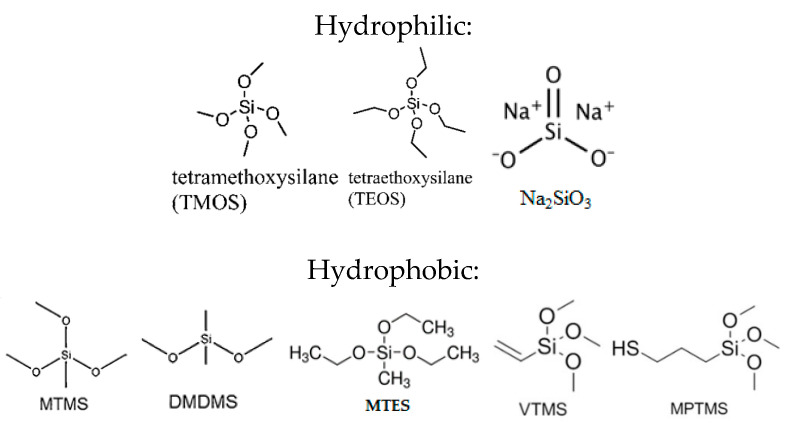
Common precursors for silica aerogels applied in CO_2_ capture.

In the sol-gel process, the precursor is dissolved in the solvent (water and/or alcohol) and an acidic and/or basic catalyst and transformed into a colloidal gel structure by a set of hydrolysis and polycondensation reactions which govern the final properties of the gel [15]. Precursors for aerogel must be soluble during the reaction and must be as reactive as possible to be able to participate in the gel-formation process. The flexibility and superhydrophobicity of silica aerogels are significantly important for their long-term applications [16]. Tetraethoxysilane (TEOS) is one of the most popular precursors deployed extensively to produce silica aerogels. Tetramethoxysilanes (TMOS) have served as starting materials for sol-gel precursors for silica aerogels, and undergo more rapid hydrolysis than TEOS [17]. Wang et al. compared the two most common silica aerogel precursors and found that TMOS yields narrow and uniform pores and higher surface area than TEOS [18]. Due to the highly porous properties of aerogels, they tend to collapse due to their mechanical properties. Therefore, there has been an attempt to prepare aerogels with improved mechanical properties for unbroken silica aerogel products. For instance, methyltriethoxysilane (MTES) was used by Colon et al. (1996) for improving the flexibility and mechanical strength of the aerogel network produced from its and higher surface area than TEOS [19]. Harreld et al. [20] found that adding methyltrimethoxysilane (MTMS) to TMOS or MTES to TEOS improves the hydrophobicity of the aerogel and shifts the pore-size distribution towards larger pore radii [19]. Nadargi et al. prepared flexible superhydrophobic silica aerogels using a MTES followed by supercritical drying [21]. They modified the surface of aerogels by trimethylchlorosilane (TMCS) to achieve the hydrophobic silica aerogel [21]. The most common hydrophilic and hydrophobic precursors for silica aerogels applied for the purposes of CO_2_ capture are listed in Figure 2.

**Scheme 1:**
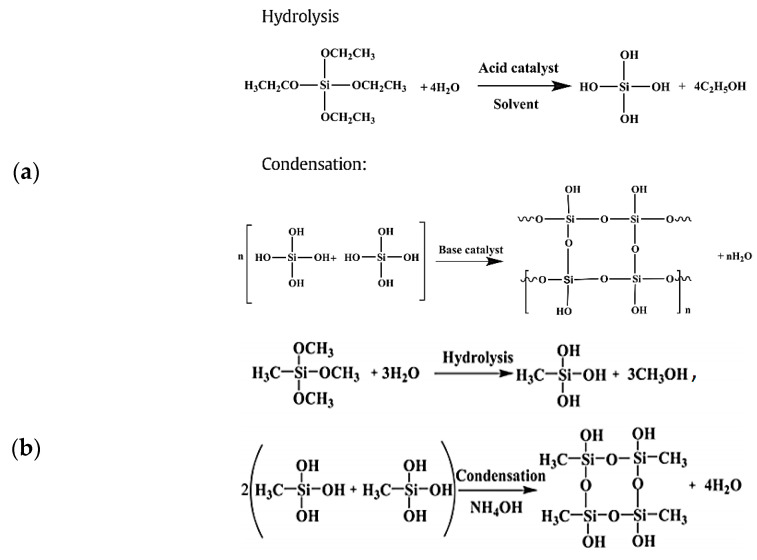
The hydrolysis and condensation mechanism of TEOS (**a**) and MTMS (**b**) precursors in an aqueous phase.

The sol-gel processes for hydrophilic (TEOS) and hydrophobic (MTMS) silica aerogels as a result of the hydrolysis and condensation mechanism are shown in Scheme 1a [22] and Scheme 1b [23], respectively.

Yun et al. produced silica aerogel microspheres with a low density (0.08 g cm^−3^) and a high porosity (96%) via APD using MTMS as a silica precursor [23]. As shown in Scheme 1b, the MTMS effectiveness—as a precursor—hinges on a non-hydrolyzable methyl group (–CH_3_), which serves to eliminate the irreversible shrinkage and capillary pressure without solvent exchange and further surface modification; tis renders this process highly suitable for large-scale production. However, the silicon alkoxides used as a precursor are neither environmentally friendly nor inexpensive at an industrial scale. As a cheap precursor, sodium silicate constitutes a proper alternative for alkoxides as the starting material for silica aerogels [24]. Lin et al. coated sodium silicate-derived silica aerogels on Al_2_O_3_ membranes featuring further hydrophobic surface modification by fluoroalkylsilane (FAS) [24]. Owing to their low-cost and high CO_2_-capture capacity, their sodium silicate-derived hydrophobic silica aerogels have illustrated good feasibility for applyication in large-scale CO_2_ absorption and in industrial power plants.

**Scheme 2:**
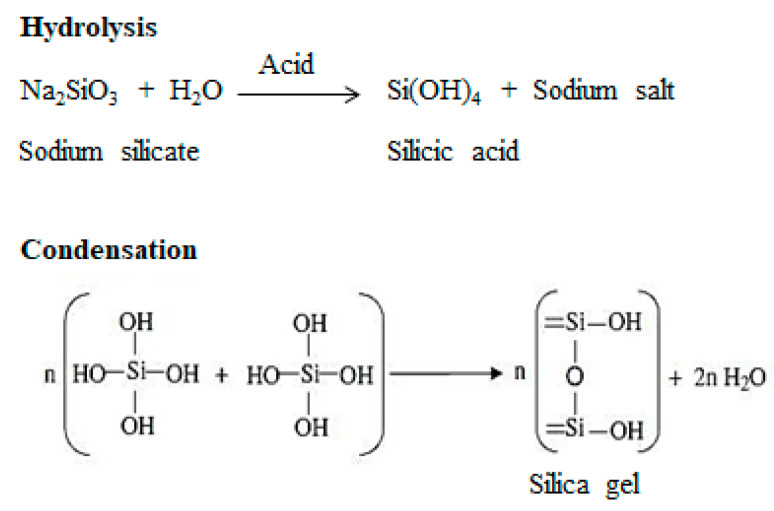
Chemical equations for the synthesis of a silica gel from Sodium silicate.

Sarawade et al. produced sodium silicate-based hydrophobic aerogels with Na_2_SiO_3_ as a precursor and surface modification using trimethylchlorosilane (TMCS) by an ambient-pressure drying process [25]. The hydrolysis and condensation of silica aerogel from sodium silicate occurred are shown in Scheme 2.

**Figure 3:**
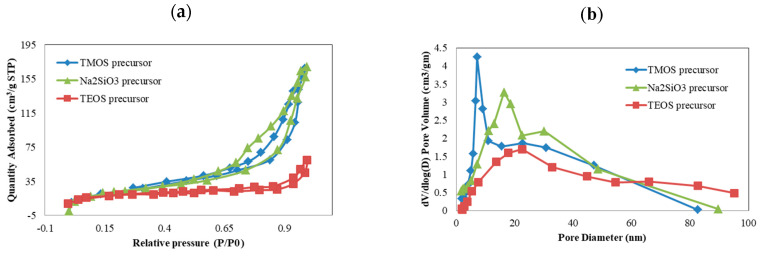
Adsorption–desorption isotherms (**a**) and pore-size distribution (**b**) for TMOS-, TEOS-, and Na_2_SiO_3_-based silica aerogels.

Pisal and Roa compared the properties of various precursors (TEOS, TMOS, and Na_2_SiO_3_) of silica aerogels [26]. They found that the surface area of the Na_2_SiO_3_-based aerogel was larger than the TMOS-based aerogel, and the TEOS-based aerogel features the smallest surface area. Figure 3 shows that all of the three precursors have mesoporous structures; however, the Na_2_SiO_3_-based aerogel sports a more consistent pore-size distribution [26]. Figure 3b shows the adsorption-desorption isotherms of TEOS-, TMOS-, and Na_2_SiO_3_-based aerogels belonging to mesoporous materials, together with the probable presence of slit-like inter-particle pores [26].

**Figure 4:**
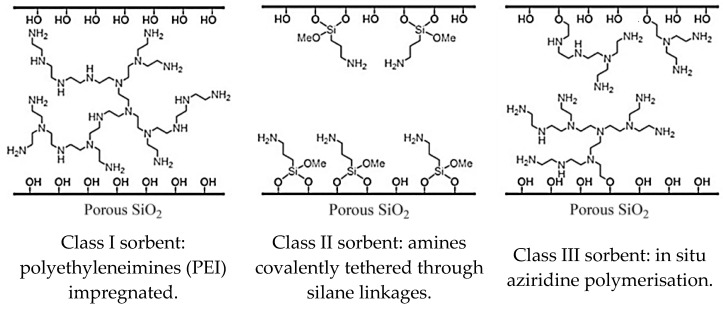
Three classes of amine-modified sorbents.

Materials prepared from the sol-gel method generally have internal and external surfaces covered by final hydrolyzed groups, which can be replaced by other functional groups using hydrolyzable molecular precursors which contain the preferable functionality [27]. Modification of aerogels with amine compounds improves CO_2_-sorption capacity and selectivity by combining the reaction properties of amine species (high CO_2_ selectivity) and the porous structure of silica aerogels (high surface area) [2]. Therefore, amine-modified aerogels have much higher CO_2_-capture capacity [28] compared with unmodified silicas-based ones. Schematic illustrations of three classes of amine-modified sorbents are shown in Figure 4.

**Scheme 3:**
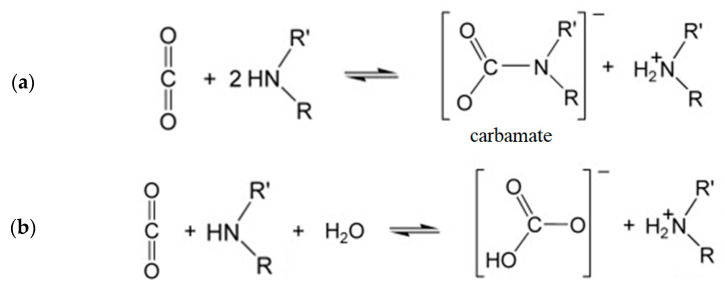
Interaction mechanism between CO_2_ and amino groups (**a**) under dry condition, (**b**) under humid condition.

CO_2_ capture by amines in solid adsorbents under humid or dry conditions follows similar reaction mechanisms. Carbamates can form by a reaction between a primary/secondary amine and CO_2_ [2,11]. As shown in Scheme 3, for each CO_2_ molecule, two amine functional groups require under dry conditions, one as a proton receptor and the other as a carbamate anion (a) and one amine functional group is required in the presence of water (b).

**Scheme 4:**
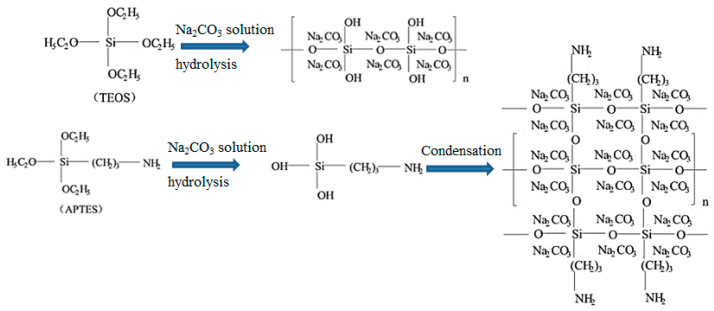
Hydrolysis and condensation of amine-modified sodium-based aerogel.

Yu et al. prepared an aerogel using Na_2_CO_3_, 3-aminopropyltriethoxysilane (APTES), and TEOS with a sol-gel method to evaluate the CO_2_-sorption capacity in a fixed-bed reactor [29]. The synthesis of the sodium-based aerogel is illustrated in Scheme 4. First, TEOS and APTES were hydrolyzed by water in Na_2_CO_3_ solution. The sol was then aged and turned into gel, along with the formation of -O-Si-O- structure. Meanwhile, the active ingredients Na_2_CO_3_ and -NH_2_ were distributed uniformly onto the material framework.

Theoretically, all the Na_2_CO_3_ and amine on sorbents could have been used for CO_2_ sorption through R1 and R2 in the presence of water according to the following reactions [29]:

R1: Na_2_CO_3_ + H_2_O + CO_2_ → 2NaHCO_3_

R2: -NH_2_ + H_2_O + CO_2_ → -(NH_3_)^+^(HCO_3_)^−^

Due to the non-equal distribution of active ingredients on the surface of the sorbents, the theoretical sorption capacities were not reachable. Loading optimum Na and N content is significant for increasing CO_2_-capture capacity [29].

**Figure 5:**
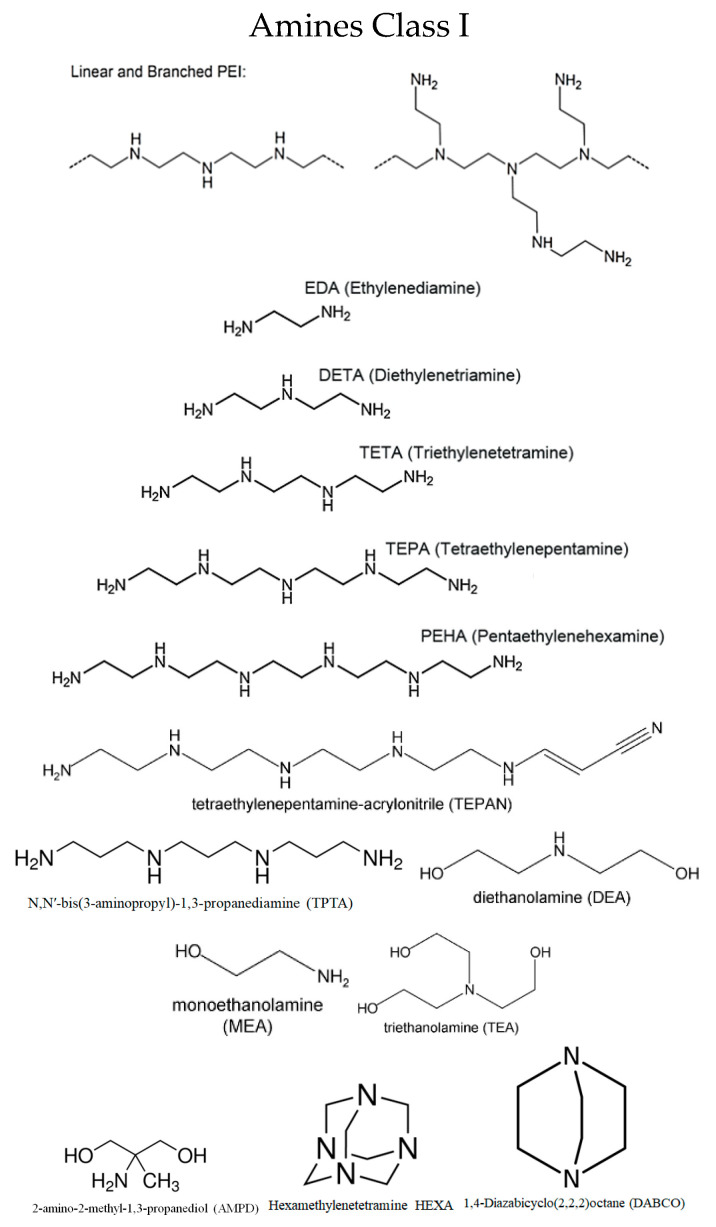

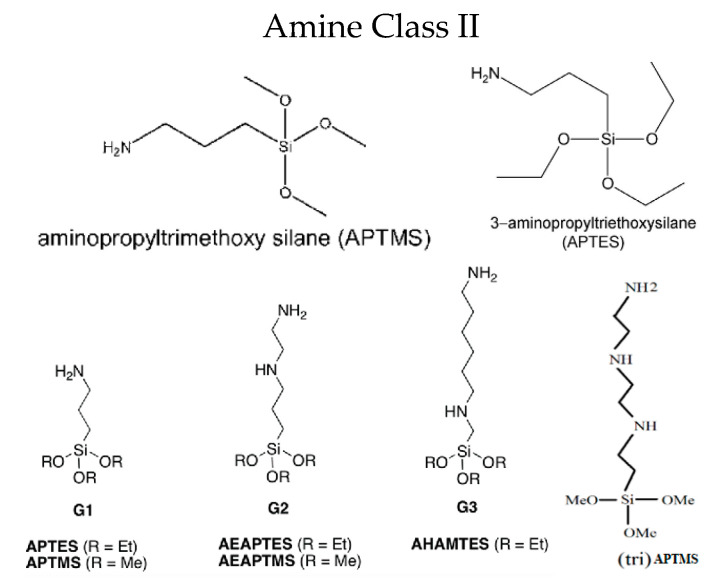
Common amines used for aerogels applied for CO_2_ capture.

The common amines used in the modification of aerogels for CO_2_-capture applications are shown in Figure 5.

Owing to the high N-atom concentration of PEI and TEPA (33% N content for PEI and 37% for TEPA) and their high boiling point, these are the two most widely Class I amines studied for CO_2_ capture [30]. PEI exists in several forms, linear and branched as shown in Figure 5. Kishor and Ghoshal reported that despite the specific surface area of silica aerogel significantly decreased by 93.3% after loading PEI into their aerogel, their prepared PEI-impregnated silica aerogel showed a high CO_2_-adsorption capacity. The advantage of physically-absorbed amines to the aerogel surface is that they achieve a greater total CO_2_-absorption capacity compared to the surface modified with chemical linkage; however, they have lower cyclic stability due to the evaporation of the amine during the thermal regeneration process [30]. Polyethylenimine (PEI) is the most used amine that physically impregnates porous silica, owing to its high amount of primary and secondary amine groups in PEI contributes a lot to the adsorption capacity. However, if the PEI concentration is too high, the pores may be blocked by PEI and reduces the CO_2_-capture capacity, as mentioned previously. Fang et al. examined the effect of additive on performance of PEI modified silica aerogels and found among their tested aerogels, a doped SA-PEI-N-Y loaded with 45% PEI and 15% N-Y (N-[3-(Trimethoxysilyl)propyl]ethylenediamine, denoted as N-Y) displayed the highest CO_2_ (1.8 mmol g^−1^). Amine-modified SiO_2_ aerogels using PEI, and TEPA [31,32], have been developed with a high CO_2_-adsorption capacity of 6.97 mmol∙g^−1^ (in 10% CO_2_ + N_2_). In another study, Wang et al. prepared PEI-modified silsesquioxane aerogels which obtained an adsorption capacity of 3.3 mmol∙g^−1^ from pure CO_2_ [33].

Functionalizing aerogels with tetraethylenepentamine (TEPA) enhances the capacity of CO_2_ take-up significantly due to its rich amino groups as well as its low molecular weight, which makes it facile to reach all of its pores. Linneen et al. developed silica aerogels functionalized with tetraethylenepentamine (TEPA) which have a CO_2_-adsorption capacity of 3.5 mmol∙g^−1^ at 75 °C [6,34]. Wang et al. developed sorbents by physical impregnation of TEPA into silica aerogels, which obtained an adsorption capacity of 4.28 mmol∙g^−1^ [35]. TEPA may be the best amine in terms of adsorption capacity, but it is thermally unstable and evaporates during regeneration cycles of CO_2_ adsorption [36].

**Scheme 5:**
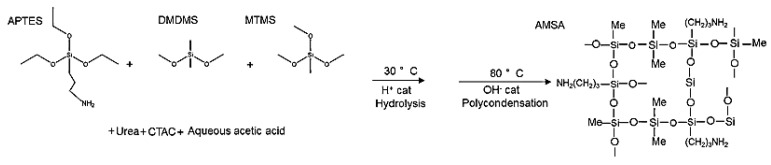
One-pot acid-base sol-gel synthesis for the AMSA [37].

The most commonly used amino silane for modifying silica aerogels is 3-aminopropyltriethoxysilane (APTES). Fan et al. in 2015 employed a MTMS, DMDMS and APTES co-precursor via a (one-pot) co-condensation method to prepare AMSAs (Scheme 5). This kind of silica aerogel, combined with extraordinarily flexibility, high CO_2_ adsorption capacity, boasts ease of regeneration and excellent stability (structure, composition and cyclic application properties); moreover, its CO_2_-adsorption capacity can reach 6.45 mmol∙g^−1^ in simulated ambient air under 1 bar of dry CO_2_, as well as good stability over 50 adsorption–desorption cycles.

Cui et al. employed 3-aminopropyltriethoxysilane (APTES) to functionalize the surface of silica aerogels to capture CO_2_ directly from air [38]. They compared CO_2_ sorption with the unmodified silica and reported that the modified aerogel nearly tripled the adsorption capacity of CO_2_ at 25 °C. Begag et al. produced sorbents using mono-, di-, and tri-aminoalkylmethoxysilanes. The sorbent was prepared by using the precursors of TEOS and MTES modified by APTES, demonstrating the high-performance of CO_2_ adsorption of 1.4 mmol∙g^−1^ [39]. Wang et al. [33], Begag et al. [7], and Fan et al. [37] combined three precursors (APTES, dimethyldimethoxysilane, and MTMS) for preparing a flexible, mechanically robust and hydrophobic amine-functionalized silica aerogel [40]. The equilibrium CO_2_-sorption capacities of these APTES-modified aerogels ranged from 3.5 to 6.5 mmol∙g^−1^ at 25 °C. Garip scrutinized amine-treated APTES, achieving a capacity of 5.53 mmol∙g^−1^ [41].

3-Aminopropyltrimethoxysilane (APTMS) is also another commonly used amine for CO_2_ capture. The adsorption capacity of APTMS is high, owing to its high amine density [42]. Santos et al. was the first group to study the effect of modification of silica aerogels with alkanolamines on CO_2_-capture capacity [43,44]. They modified their aerogel by adding the 3-aminopropyltrimethoxysilane (APTMS) and wollastonite (CaSiO_3_). Wörmeyer and Smirnova compared the CO_2_-capture capacity of mono-amine (APTMS) and tri-amine (3-trimethoxysilylpropyl) diethylenetriamine (T-APTMS) for modification of silica aerogels and showed that tri-amine achieved about double the sorption capacity than mono-amine [45,46].

For aerogels modified by an amine, the adsorption capacity is usually proportional to the amount of loaded amine. However, an increase in the concentration of amine beyond certain limits leads to pore pluggage. Moreover, silane reagents with three alkoxy groups are prone to hydrolysis and self-polymerisation on the aerogel surface, which leads to blocking the inner pore and consequently decreasing the CO_2_ capture as the inner pore structure is likely to be blocked. Therefore, aminosilanes with two alkoxy groups could be the safer option to avoid the undesired blocking of functional amine sites [47].

**Figure 6:**
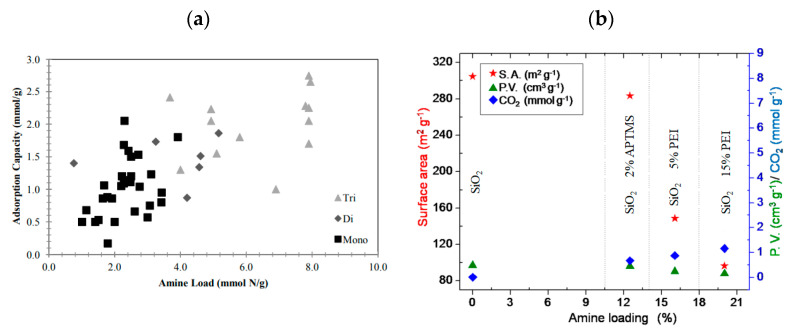
(**a**) Adsorption capacity of Mono, Di, and Tri-amine of PEI Impregnated Silica supports (all these sorbents were prepared and examined under similar conditions) [36]. (**b**) Surface properties and CO_2_ adsorption as a function of amine loading [48].

The adsorption efficiency is highly dependent on some other properties, such as surface morphology, pore size and available pore volume of the aerogel. Figure 6a indicates the larger N content of PEI can lead to higher adsorption capacity [36]. As triamine can graft more mmol of N per gram of sorbent, it has the greatest performance compared to the di- and mono-amine versions in terms of adsorption capacity. 

Minju et al. used APTMS to modify silica aerogels derived from water glass [48] and demonstrated the effect of amino loading is important for realising good selectivity and enhanced sorption capacity. They studied the effect of amine loading and its efficiency in the absorption of CO_2_ gas concerning amine functionality and pore structure of their PEI-modified silica aerogels [48]. Figure 6b summarizes the changes in surface area, pore volume, and CO_2_-adsorption capacity of silica aerogels, along with the change in amine content [48]. This figure shows that increasing amine loading leads to an increase in CO_2_-adsorption capacity. However, the pore volume and specific surface area for the aerogel modified with 15% PEI is lower than the aerogels containing 2% APTMS and 5% PEI, so that high sorption capacity was observed. Therefore, besides sorbents physical/textural properties such as a high pore volume and surface area, the effect of amine loading is significant in achieving higher CO_2_ sorption capacity.

The modification of porous support material with ionic liquids (ILs) has become the main topic of many studies due to the high CO_2_-sorption capacity of ILs. Garip and Gizli [41] added ionic liquid (1-Ethyl-3-methylimidazolium bis (trifluoromethylsulfonyl) imide) to their amine-based silica aerogels to improve CO_2_-capture performances of the prepared aerogels and achieved an adsorption capacity of 5.53 mmol∙g^−1^. Besides supported amines, supported potassium carbonate (K_2_CO_3_) has been considered as a potential sorbent for CO_2_ capture due to its low cost and high CO_2_-capture capacity. Zhao et al. prepared K_2_CO_3_-modified silica aerogels using commercialized silica aerogels [49,50]. However, they found that the sorbent showed low CO_2_-adsorption capacity (0.15 mmol∙g^−1^), due to a large amount of hydrophilic group existing in commercialized silica aerogels. Therefore, in another study, they synthesized modified silica aerogels with a sol-gel method using tetraethoxysilane (TEOS) as a precursor and carried out impregnation with K_2_CO_3_, and improved the CO_2_-capture capacity to 0.53 mmol∙g^−1^ [51].

The precursors, as well as modification groups of silica aerogels, in addition to their CO_2_ capacity as the output of aerogels mentioned in this section, are summarized in Table 1.

Based on the results from Table 1, both sorbent Classes I and II show promising results for CO_2_ capture. Although physical modification of amine is easier and usually shows better CO_2_ adsorption, after cycling and regeneration steps, there is the possibility of removing Class I amines after regeneration and illustrating poor cycling performance [2]. From Table 1, it can be observed the silicon alkoxides as a precursor for preparing silica aerogel can perform better for CO_2_ capture; however, using Na_2_SiO_3_ as silica aerogel precursor can decrease the cost of aerogel production. Between the studies listed in Table 1, the hydrophobic silica aerogel prepared by Fan et al. with high CO_2_ adsorption capacity could indeed be a promising candidate for scale up, as their aerogel was flexible featuring high mechanical strength and hydrophobic character. Moreover, their aerogel was prepared by the one-pot method, which is not only a low-cost method, but also adding APTES in the sol-gel process can largely remove the underlying process’s need for a catalyst, as the APTES itself acts as an internal catalyst.

## 3. Organic Aerogels

The term “organic aerogel” refers to many different kinds of aerogels with properties arising from the polymer, which makes an organic aerogel generally less fragile than inorganic aerogels. This type of aerogel is divided generally into two categories of synthetic and bio-based. The development of bio-based sorbent which demonstrates high CO_2_-adsorption capacity is a promising alternative vis-à-vis inorganic aerogels.

Organic aerogels can be made from Resorcinol/Formaldehyde (RF) [53,54,55,56], 2,4-dihydroxybenzoic acid (DHBA)/F [57], Phenol/Formaldehyde (P/F), Melamine/F (MF) [58], Polyurethane-Dichloromethane [59], Cresol/Formaldehyde (CF) [60], RF/CTAB [55], Pararosaniline base (PAB) and 1,3,5-triformylbenzene (TFB), Phloroglucinol-formaldehyde (FPOL) [61], Phloroglucinol-terephthalaldehyde (TPOL) [61], Poly(vinyl alcohol) PVA aerogels [62], Carbon nanotubes (CNT) [63], and Graphene [64,65,66,67,68,69,70]. Different catalysts are usually used to prepare RF aerogel [71].

Masika and Mokaya developed a simple method to prepare high-surface-area carbon aerogels (CAs) from Melamine-Formaldehyde (M/F) resins, via metal-salt (CaCl_2_) templating without any activation [58]. The metal salt acts as a porogen to generate carbon aerogels with a high surface area of up to 1100 m^2^∙g^−1^, which exhibit high CO_2_ uptake of up to 2.2 mmol∙g^−1^ at atmospheric conditions. Marques et al. obtained 2.15 mmol∙g^−1^ capacity of CAs using DHBAF and RF as precursors [57].

Carbon aerogels are organic aerogels that undergo a step of calcination and activation for sorption purposes [72]. To generate highly porous carbon materials, CAs are subjected typically to a physical or chemical activation process. Physical activation is applied by exposing the carbon precursor to high temperatures in the presence of steam or an oxidising gas (e.g., carbon dioxide). In this step, aerogel is exposed to high temperature—even higher so than pyrolysis temperatures to form the gel (usually above 600 °C)—and atmospheric pressure with/without an inert atmosphere of N_2_ or Ar to decompose some volatile materials and enhances the network with pure metal oxides [54,73]. The pyrolysis temperature can significantly alter the final properties of aerogels as the product. For instance, a lower pyrolysis temperature (e.g., 600 °C) usually increases surface area, whereas higher temperatures can decrease the surface area, and, consequently, the double-layer capacitance [74]. Liu et al. investigated the effect of calcination temperatures (600 °C to 900 °C) on the pore and surface properties of CAs prepared for CO_2_ adsorption [75]. It was found that the surface area, and, consequently, CO_2_-adsorption capacity, firstly increased by increasing temperature, and 800 °C is the optimum calcination temperature; beyond that, the surface area and CO_2_-capacity decrease with further temperature increases. 

**Scheme 6:**
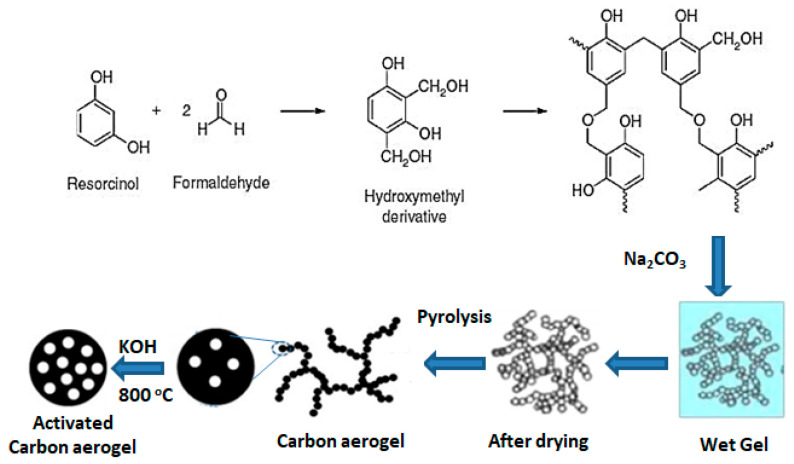
Synthesis and activation of carbon aerogel.

For chemical activation, an activating reagent impregnates onto a carbon precursor and is then subjected to high temperatures in an oxygen-free environment. For chemical activation, agents such as KOH, NaOH, are mixed with the precursors, and then heat treatment is applied in an inert atmosphere at different temperatures [76]. Comparing physical and chemical activation, the activation of gaseous activator CO_2_ is easier because no effort is required at post-treatment stage to remove residual solid activators, such as NaOH or KOH [77]. Robertson and Mokaya prepared high surface-area-activation carbon aerogels with activation at 600, 700, and 800 °C, as well as chemical activation with KOH [76]. They showed that the activation temperature and KOH/carbon ratio affect the textual properties of CAs and CO_2_ uptake. The activated CAs showed CO_2_-adsorption capacity between 2.7 and 3.0 mmol∙g^−1^ under flowing pure CO_2_ and atmospheric conditions. Scheme 6 illustrates the synthesis and activation of carbon aerogel from RF as the most common precursor used in CAs.

Although the abovementioned activation methods only affect the textual properties of carbon aerogel, modifying CAs by amines has also proven to be promising for achieving effective CO_2_ adsorption [28]. Oxidation, and other side reactions, may occur that affect the performance of these aerogels due to amine volatilisation and urea formation. The maximum amine loading in the impregnation of carbon aerogels can be higher than 85% wt, without blocking the pores for CO_2_ diffusion into the bulk of the adsorbent [63].

**Scheme 7:**
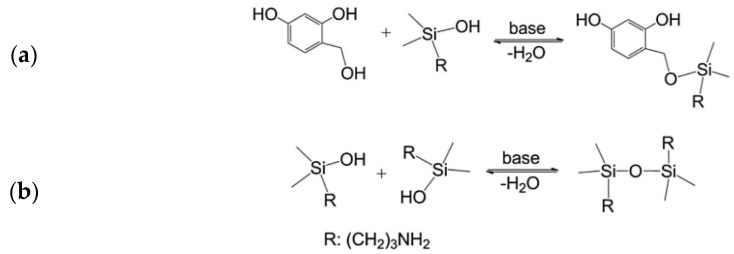
Mechanism of the reaction between RF and APTES under base condition [78]. (**a**) the hydrolysed species of APTES (Si-OH) reacts with hydroxymethylated R; (**b**) Si-OH of APTES reacts with each other.

In the sol-gel reaction of RF aerogel, APTES can play the role of internal catalyst not only to drive the sol-gel reaction but also for the reactant to form a gel framework/network. Reactions between APTES and RF are shown in Scheme 7. Under basic conditions, building Si–O–C linkage, the hydrolysed species of APTES (Si-OH) reacts with hydroxymethylated R (Scheme 7a). To build Si–O–Si linkage, Si-OH of APTES could react with each other (Scheme 7b) [78]. 

Marques used ten different amines (2-Amino-2-methyl-1,3-propanediol (AMPD), Ethanolamine, Pentaethylenehexamine, Tetraethylenepentamine TEPA, N,N′-bis(3-aminopropyl)-1,3-propanediamine TPTA, Triethylenetetramine TETA, Diethylenetriamine DETA, Polyethylenimine, ethylenediamine branched PEI, Hexamethylenetetramine,1,4-Diazabicyclo(2,2,2)octane) modified onto two types of carbon aerogel to analyse the capacity of each sample for CO_2_ desorption [8]. Gromov et al. prepared a series of ultra-light aerogels with mechanical, chemical, and thermal stabilities of oxidized carbon nanotubes and cross-linked polyvinyl alcohol, and examined them as amine impregnated solid supports for CO_2_ capture [63]. Their PEI-impregnated aerogels demonstrated CO_2_ capacity of 3.3 mmol∙g^−1^ in a dilute gas stream. In another study done by Chen et al., TEPA was used for modifying CAs, which demonstrated high CO_2_ adsorption of up to 4.1 mmol∙g^−1^ at 75 °C under 10 v% CO_2_/N_2_ flow [79]. Moreover, amine modification of CNF aerogels could improve CO_2_-adsorption capacity, owing to the abundance of hydroxyl groups on CNF. For example, the aminated nanocellulose aerogel prepared by Zhang et al. achieved an adsorption capacity of the modified nanocellulose aerogel for CO_2_ (1.59 mmol∙g^−1^), increasing by 7.4 times compared with that of the pristine one (0.19 mmol∙g^−1^). Yang et al. prepared CA–K_2_CO_3_ nanocomposites by impregnation of K_2_CO_3_ nanocrystals into CAs [80]. Their CA–K_2_CO_3_ aerogel prepared from a CA with 7 nm pores exhibited CO_2_ capture capacity of 2.45 mmol∙g^−1^. Xie et al. synthesized CAs, activated with KOH and amine modification (PEI and TEPA). The highest adsorption capacity of 2.06 mmol∙g^−1^ observed for 55 wt.% (PEI)-loaded on activated CA was at the activation mass ratio KOH to CA of 1.

There is a good deal of research on the incorporation of nitrogen atoms into the surface of pores to enhance the adsorption capacity of CA. Ello et al. prepared CAs from a nitrogen-containing polymer precursor using urea as a nitrogen source into the polymer [53]. They showed that the N-doped carbon aerogel has a different structure from the non-N-doped carbon aerogel, and, consequently, adsorbs more CO_2_. Their developed N-doped CA achieved CO_2_-adsorption capacity up to 3.6 mmol∙g^−1^ at atmospheric conditions. Jeon et al. prepared N-doped CAs with CO_2_ capture capacity of 2.68 mmol∙g^−1^ (at resorcinol/melamine = 1:0.3) [81]. They found the amount of melamine plays a crucial role in determining the CO_2_-capture capacity as further increasing nitrogen content (with introducing the melamine), decreasing the surface area and the pore volume of CAs due to occupation of pore spaces by nitrogen.

Although these previous studies demonstrate promising results for adsorption of CO_2_, there are still some limitations for application on large scales. Their precursors are commonly not from renewable sources and their production is costly [82]. Therefore, sustainable and biomass-derived products, such as lignin, alginate, protein, chitosan [65], cellulose [83], as bio-based organic precursors for CO_2_ sorbents have recently attracted great attention due to their renewable and environmentally friendly nature. 

Zhuo et al. produced a porous cellulose aerogel with a high specific surface area and excellent super capacitance [77]. Their cellulose-derived hierarchical porous carbon aerogel showed a good CO_2_-adsorption capacity of 3.42 mmol∙g^−1^ at ambient pressure and temperature. Miao et al. have used the old corrugated containers (OCCs) as raw materials to synthesize a series of aerogels with varying cellulose concentrations in NaOH/urea solution via a freeze-drying process. To produce purified cellulose, they treated OCCs in KOH, lignin and hemicellulose. The resulting aerogels had a rich porous structure with a CO_2_ capture capacity of 1.96–11.78 mmol∙g^−1^ at ambient temperature and pressure.

For aerogels derived from cellulose, there is low carbon content and the absence of aromatic structures in the precursor, which limits scope achieving a high capacity of CO_2_. Therefore, developing new precursors for carbonaceous aerogels from environmentally sustainable and renewable and sustainable sources featuring tunable structure is required as a CO_2_ sorbent. Geng et al. developed a new type of carbon aerogel (LTCAs) with a hierarchical anisotropic porous structure derived from kraft lignin/TOCNF precursors (extracted from softwood powder) which demonstrated CO_2_-adsorption capacity up to 5.23 mmol∙g^−1^ at atmospheric conditions [82]. They controlled the structure of their CAs by adjusting carefully the weight ratio of lignin to cellulose nano-fibres in the precursors, which affect their final porosity and surface area. 

Table 2 synopsizes the various studies mentioned in this section, including their precursors, activation methods and, of course, CO_2_-adsorption capacity.

Amongst all organic aerogels listed in Table 2, the etched CAs prepared by Majedi far et al. show the highest CO_2_-sorption capacity up to 14.8 ± 3.9 mmol∙g^−1^ due to their extremely high surface area created by etching [61]. However, between the organic aerogels, bio-based aerogels such as celluloses have recently attracted much attention with excellent properties, such as being environmentally friendly, renewable, biocompatible, biodegradable, as well as being inexpensive.

## 4. Hybrid Aerogels

Hybrid aerogels are materials, the structures of which are formed by two solid phases of different chemical compositions that interact with each other at the molecular level via strong covalent links—for example, mixtures of inorganic and organic moieties. Hybrid aerogels represent an attractive and novel class of aerogels that inherit from the individual components and can allow for applications that would hardly be possible with only a single component. Hybrid aerogel organic-inorganic bridged alkoxysilanes can improve the mechanical performance of aerogels [22]. At first, hybrid aerogels were advanced based on isocyanate-crosslinked vanadia aerogels [86], CuO resorcinol–formaldehyde aerogels [87], and epoxy/polystyrene-linked SiO_2_ aerogels with upgraded mechanical properties [88]. For hybrid aerogels, successful mixtures can be obtained with organic polymers capable of forming covalent or hydrogen bonding [89]. Indeed, one important direction in predictive materials design, which may be set to allow for the systematic appraisal of different types of chemical bonding, lies in preliminary computer-aided materials design leverages Density Functional Theory, which allows for careful and systematic comparison of families of promising molecular scaffolds and appraisal of all types of chemical/physical bonding in materials at different levels of DFT treatment and judicious comparison and optimisation with experiment [90,91].

**Scheme 8:**
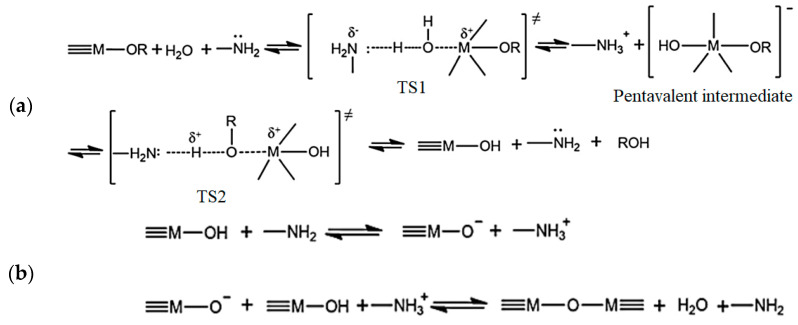
Hydrolysis (**a**) and Condensation (**b**) mechanism of the sol-gel reaction (M = Si or Ti) [95].

Kong et al. proposed a series of hybrid aerogels modified by amine with a one-step preparation method which is simple, cost-effective and demonstrated promising CO_2_ capture. They developed an amine hybrid resorcinol-formaldehyde/silica composite aerogel (AH-RFSA) with a facile method which showed a CO_2_-adsorption capacity of 1.80–4.43 mmol∙g^−1^ [92]. By using the resorcinol, formaldehyde, anhydrous sodium carbonate, they developed a new type of hybrid aerogel (AFMSiCA) [93]. Their amine-functionalized monolithic silicon-carbide aerogels were tested as sorbent and showed CO_2_ uptake of 1.81 mmol∙g^−1^. In another study, they synthesized amine hybrid silsesquioxane aerogel hybrid aerogels via a one-pot method using TEOS as a precursor and APTES as a modification group (AHSA) [94]. In reaction, TEOS is much slower than APTES, and thus affects negatively the surface amine content of amine hybrid silica aerogel. To compensate for that, in another study, they used tetra-n-butyl titanate (TBT), a more reactive transition metal-based precursor along with APTES to synthesize amine hybrid titania/silsesquioxane composite aerogel (AHTSA) as a CO_2_ adsorbent [95]. In their one-pot method to prepare AHTSA hybrid aerogel, TBOT/APTES/EtOH/W was used without requiring any catalyst, because APTES acted as an “internal catalyst” in the sol-gel process [95]. According to Scheme 8, APTES controls the hydrolysis and condensation steps due to the nucleophilic activation of Si and Ti atoms by the N lone pair [95]. In the hydrolysis step, first, TBOT and APTES make an unstable transition state TS1 and TS2 and at the end, M–OH was formed by leaving alcohol. In the condensation step, by assisting -NH_2_ as the catalyst, the anion intermediate (≡M-O^−^) attacks Si or Ti atom of another hydrolyzed species to builds M–O–M linkages [95] (Scheme 8).

In another investigation, they prepared a new amine hybrid zirconia/silsesquioxane composite aerogel (AHZSA) from mixing the Zirconium(IV) n-propoxide as a precursor with APTES, which showed a CO_2_-capture capacity of 2.7 mmol∙g^−1^ [96].

**Scheme 9:**
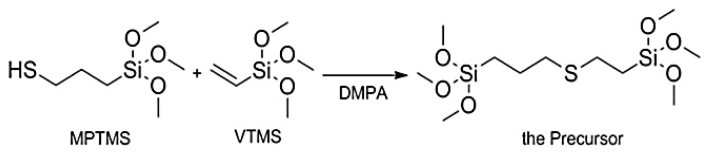
Synthesis of the precursor from MPTMS and VTMS [33].

Wang et al. prepared an inorganic-organic hybrid aerogel from sol-gel polymerisation of bridged silane precursors [33]. As shown in Scheme 9, silsesquioxane precursor from (3-mercaptopropyl)trimethoxysilane (MPTMS) and vinyltrimethoxysilane (VTMS) were formed. Improved mechanical properties and the facile, low-cost preparation procedure (vacuum-drying) provide the possibility of applications of this aerogel for CO_2_ capture.

Nanocellulose−silica aerogel can be prepared by the two-step gelation−impregnation process or one-step homogeneous dispersion and simultaneous gelation of nanocellulose and silica precursor such as TEOS and methyltrimethoxysilane. Jiang et al. 2018 use CNFs/Na_2_SiO_3_ composition as precursors and freeze-dried produced hybrid aerogel with ultralow density, high specific surface and pore volume with the capability of capturing CO_2_ with an adsorption capacity of 1.49 mmol∙g^−1^ [97]. Alhwaige et al. [98] synthesized chitosan aerogels with graphene-oxide nanosheets. A CO_2_-capture capability of up to 4.14 mmol∙g^−1^ was observed by using their hybrid aerogels. The same group reported CO_2_-adsorption capacity of up to 5.72 mmol∙g^−1^ at atmospheric conditions achieved by their CAs prepared from biobased chitosan-polybenzoxazine (CTS-PBZ) and sodium montmorillonite (Na-MMT) as a precursor [85].

Table 3 shows that similar to silica aerogels and carbon aerogels, amines could be used for modification of hybrid aerogels, and, although less studied at present, this represents a promising future avenue of research.

## 5. Conclusions

The application of aerogels to capture CO_2_ is quite promising, owing to their desirable properties, such as high porosity, high surface area, and low density. Amongst all types of aerogels, silica, carbon, polymer, and natural-based (i.e., cellulose) aerogels are mostly applied for environmental cleaning and capturing CO_2_, owing to their distinctive physicochemical properties. Considering the versatility of sol-gel processing, the microstructural patterns, composition, and surface chemistry of aerogels can be tailored by sol-gel parameters and different types of precursors for applications of CO_2_ capture. Therefore, the selection of the appropriate precursor and optimum parameters are very important to ensure the quality of aerogels. For silica aerogel organosilane (co)precursors, such as methyltri(m)ethoxysilane (MTES, MTMS), dimethyldi(m)ethoxysilane (DMDES, DMDMS), and also trimethylethoxysilane (TMES) as hydrophobic precursors, these not only increase the life of aerogels but also, importantly, enable ambient-pressure drying as a potentially more cost-effective alternative to supercritical drying. Moreover, they minimize the thermal energy required for the regeneration of aerogel as a CO_2_ adsorbent. As a low-cost alternative precursor for silica aerogel, sodium metasilicate is a good candidate suitable for large scale applications. Although traditional aerogels illustrated promising results for CO_2_ capture, recently there has been more interest in renewable carbon resources with adjustable structures that are environmentally sustainable with remarkable performance in the relevant applications. Accordingly, recent investigations have focused on using aerogels as new CO_2_-capture materials by controlling the chemo-physical properties of different types of aerogels. New advanced materials stemming from recent and exciting developments in materials science provide new opportunities for growth prospects through the support of new existing industrial and commercial products and processes. Indeed, from the materials perspective, hybrid materials with the possibility of combinations of different polymer matrices and synthesising hybrid aerogels with combinations of non-aerogel materials (e.g., fibres, particles, textiles) offer a promising candidate for aerogel in the application of CO_2_ capture.

Generally, amines are the most used modification group, enhancing the sorption capacity of CO_2_ sorbent, and the amine types and content of aerogels determine the sorption performance of amine-modified aerogels. Functionalization of the surface can be achieved by physically mixing; however, this method is limited because the involved molecules can be washed off the surface. Employing a chemical reaction, more sophisticated and selective surfaces can be obtained. In a one-pot method—a simple and environment-friendly method—the amine can perform the role of catalyst for the sol-gel process. For many CO_2_-capture approaches, recent studies have indicated that ionic liquids have attracted attention for absorbing CO_2_ by either physical or chemical absorption. This may be expected to provide different and diverse advantages for future aerogel studies, and represents an important new direction.

Aerogel research is still developing and enjoying significant activity now. It is to be hoped that this will motivate more researchers to focus on the preparation of novel single-component aerogels towards the ultimate goal of ambitious materials design of composite aerogels and their applications in CO_2_ capture.

## Figures and Tables

**Table 1:** CO_2_ capture by amine–silica aerogels.

Precursor	Amine Type, Amine Conc. [wt.%]	Modification Method	Adsorption Capacity [mmol∙g^−1^] & Adsorption Conditions	References
TEOS	TEPA, 80wt.%	Impregnation	6.1 at 25 °C and 1 bar	[6]
TEOS	mono, di, and tri-amine trialkoxysilanes	Grafting	0.67–1.64 at 25 °C, 100% CO_2_, 1 bar)	[52]
TEOS	TEPA, 80wt.%	Impregnation	5.1 at 75 °C under a dry 10% CO_2_/Ar stream	[34]
TEOS (APTES and TEOS ratio:1:2)	APTES	Grafting	1.95 at 25 °C and 1 bar	[38]
TMOS	APTMS 2 wt.%/ PEI 15 wt.%	Grafting/ Impregnation	0.67 1.16 at 50 °C	[48]
TMOS	APTMS, 1.44 wt.%	co- condensation & post treatment	0.523 at 250 Pa	[45]
TEOS	Mono-APTMS Tri-APTMS	Grafting	0.52–1.07 at 250 Pa moist air	[46]
MTMS (MTMS and APTES:1:1), DMDMS 4 nmol	APTES: 1 mol ratio	One-pot	6.45 at 1 bar of dry CO_2_	[37]
TEOS	APTES, 24 wt.% + IL, 28 wt.%	One-pot	5.53 at 25 °C and 1 bar	[41]
TEOS	PEI, 45wt.%	Grafting	1.8 at 25 °C and 1 bar	[11]
TEOS, Na_2_CO_3_ (mole ratio:1:1)	APTES: 1 mol ratio	Direct	2.51 at 50 °C (H_2_O/CO_2_ = 1:1)	[29]
Na_2_SiO_3_ coated on Al_2_O_3_ membrane	Fluoroalkylsilane (FAS)	Grafting	1.5 at 25 °C and 1 bar	[24]

**Table 2:** CO_2_ capture by organic aerogels.

Precursor	Carbonization/Activation	Adsorption Capacity (mmol∙g^−1^)	References
RF	Carbonized at 1050 °C	6.43 at 0 °C, 1 bar	[56]
RF/CTAB	Carbonized at 800 °C	4.19 at 0 °C and atmospheric pressure	[75]
DHBA/F	AMPD	1.59 at 35 °C and atmospheric pressure	[8]
PAB/TFB	N-doped Pyrolysis at 1000 °C	6.1 at 0 °C, 1 bar, 33.1 at 50 °C, 30 bar	[84]
RF	Activation at 800 °C (KOH carbon ratio of 5)	2.7–3.0 at 25 °C, 1 bar	[76]
MMT-CTS- PBZ	Pyrolysis at at 800 °C	5.72 at 25 °C, 1 bar	[85]
RF	N-doped, Melamine	2.68 at 25 °C, 1 bar	[81]
carbon nanotubes/PVA	75–83% PEI	3.3 ± 0.3 at 25 °C, 1 bar	[63]
PF, RF, FPOL and TPOL	Pyrolytic at 800 °C Etching at 1000 °C	14.8 ± 3.9 at 25 °C, 1 bar	[61]
DHBA/F RF	Carbonisation at 800 °C	1.2–2.14 at 25 °C, 1 bar	[57]
RF	N-doped, Urea Carbonisation at 800 °C	3.6 at 25 °C and 4.5 at 25 °C, 1 bar	[53]
Melamine/F	Activation at 800 °C KOH/carbon ratio = 2	2.2 at 25 °C, 1 bar	[58]
Cellulose	N-doped, Urea Activation at 800 °C	3.42 at 25 °C, 1 bar	[77]
RF	Impregnation of K_2_CO_3_ Carbonisation at 900 °C	2.45 at 25 °C, 1 bar	[80]
RF/CTAB	Activation at 900 °C KOH (ratio to CA: 1), 55 wt.% PEI 60 wt.% TEPA	2.06–2.84 at 75 °C, 1 bar	[55]

**Table 3:** CO_2_ capture by hybrid aerogels.

Precursor	Activating Group	Adsorption Capacity (mmol∙g^−1^)	References
CTS–GO (hybrid monolith aerogels of chitosan-Graphene oxide) aerogel	-	1.92–4.15	[98]
AHSA (Monolithic + SiC)	APTES	2.69 at 30 °C	[94,99,100]
AHTSA titania + silsesquioxane	APTES	4.19 at 30 °C	[95]
AHZSA Zirconia + Silica	APTES	2.7 at 30 °C	[96]
AH-RFSA	APTES	3.57 at 30 °C	[78,92]
AFMSiCA	APTES	1.81 at 25 °C	[93]
CNT + PVA	APTES	3.3 at 25 °C	[63]
MMPTMS, VTMS	PEI 0.35 mol∙L^−1^, Impregnation	3.3 at 25 °C	[33]
